# Dynamic analysis of CD127 expression on memory CD8 T cells from patients with chronic hepatitis B during telbivudine treatment

**DOI:** 10.1186/1743-422X-7-207

**Published:** 2010-08-31

**Authors:** Guocai Lv, Linjung Ying, Wen-Jiang Ma, Xi Jin, Lin Zheng, Lanjuan Li, Yida Yang

**Affiliations:** 1 State Key Laboratory for Diagnosis and Treatment of Infectious Diseases, Department of Infectious Diseases, First Affiliated Hospital, School of Medicine, Zhejiang University, Hangzhou, Zhejiang 310003, P. R. of China

## Abstract

**Background:**

Accumulating evidence supports the theory that expression of CD127 on CD8 T cells during the process of antiviral immune response indicates a subset of effect CD8 T cells that successfully develop into fully protective memory. CD8 T cells expression of CD127 may be used as a predictor to evaluate disease status in chronic viral infection. The aim of this study was to investigate the CD127 expression level on different subsets of CD8 T cell and explore the relationship between CD127 expression on CD8 memory T cells and serum hepatitis B virus (HBV) DNA and hepatitis B e antigen (HBeAg) levels in patients with chronic hepatitis B (CHB). We also aimed to investigate the CD127 expression pattern on CD8 memory T cells of CHB patients who were treated with Telbivudine.

**Methods/Results:**

Twenty HBeAg-positive CHB patients were selected and treated with telbivudine 600 mg/day for 48 weeks. The memory CD8 T cells were characterized by expression of CD45RA and CD27 markers. CD127 expression on the CD8 T-cell surface was measured by four-colour flow cytometry. Our results showed that CD127 expression on memory CD8 T cells was reduced in CHB patients. There was a strong negative correlation between CD127 expression on memory CD8 T cells and serum HBV DNA and HBeAg levels in CHB patients. Moreover, successful antiviral therapy increased CD127 expression on CD8 memory T cells as well as on HBV-specific CD8 T cells in CHB patients.

**Conclusion:**

These results suggest that diminished CD127 expression on CD8 memory T cells of CHB patients is a potential mechanism explaining cellular immune function impairment in CHB infection, and that CD127 expression on CD8 memory T cells is a useful indicator for evaluating the effects of anti-HBV therapy.

## Introduction

Chronic hepatitis B virus (HBV) infection remains a serious global health problem. It affects approximately 350 million people worldwide and more than 130 million in Chian[1]. It is widely accepted that the adaptive immune responses, particularly the cellular immune response, mediate clearance of HBV. Unfortunately, in most patients, chronic HBV infection leads to severe abnormalities of CD8 T-cell function, as shown by a low level of antiviral cytokines and impaired cytotoxic T-lymphocyte (CTL) activity [2].

Naive CD8 T cells that encounter their cognate antigen undergo a complex process of maturation and differentiation that ultimately leads to the generation of long-lived memory CD8 T cells, which mediate immune production from subsequent challenge with the same antigen [3]. Memory CD8 T cells are characterized by their abilities to survive homeostatically in the absence of antigen and proliferate vigorously upon antigenic re-encounter. Memory CD8 T cells are easily activated upon antigen rechallenge, in which situation they quickly produce antiviral cytokines or cytotoxic molecular [4,5].

Interleukin (IL)-7 signalling is essential to CD8 T-cell proliferation and function. The IL-7 receptor (IL-7R), a heterodimer, is composed of a unique α chain (CD127) and a common γ chain (CD132) [6]. During viral infection, CD127 expression on CD8 T cells occurs only when the antigen load is contained and sufficient CD4 T-cell help is available [7]. Persistent viral antigen load suppresses CD127 expression on primed T cells and correlates with exhaustion of a previously stable primed T-cell population [8]. Studies on patients with acute HBV infection showed that CD127 expression on HBV-specific CD8 T cells increased markedly after viral clearance [9].

In the present study, we demonstrated that CD127 expression on CD8 memory T cells was reduced in patients with chronic hepatitis B (CHB). There was a strong negative correlation between CD127 expression on CD8 memory T cells and serum HBV DNA and hepatitis B e antigen (HBeAg) levels in these patients. Moreover, successful antiviral therapy increased CD127 expression on CD8 memory T cells as well as on HBV-specific CD8^+ ^T cells in patients with CHB. These results suggest that CD127 expression is a potential indicator for evaluating the effects of anti-HBV therapy.

## Materials and methods

### Patients

This study was approved by the Ethics Review Committee of the First Affiliated Hospital, School of Medicine, Zhejiang University (Hangzhou, Zhejiang, China). The diagnosis of CHB was made according to the diagnostic standards from the National Program for Prevention and Treatment of Viral Hepatitis. A total of 20 HLA-A2^+ ^patients with CHB (8 women and 12 men; mean age 27 years) were enrolled in the study. Human leucocyte antigen (HLA) typing was performed using polymerase chain reaction (PCR) amplification with sequence-specific primers, and it was confirmed by flow cytometry.

Hepatitis B surface antigen (HBsAg), HBeAg, anti-HBc, anti-HBe and anti-HBs were quantified by radioimmunoassay (Abbott Laboratories, Abbott Park, IL, USA). HBV DNA was measured using the Amplicor HBV test (Roche Diagnostics, Basel, Switzerland) with a detection limit of 300 copies/mL. All patients were HBeAg positive and had never received anti-HBV therapy before.

At baseline, the average serum HBV DNA of the 20 patients was 7.8 ± 0.09 log_10 _copies/mL [median: (7.9 5.0-9.8) log_10 _copies/mL], and the serum alanine aminotransferase (ALT) was 174.6 ± 7.78 IU/L [median: 113 (99-567) IU/L]. All patients received telbivudine 600 mg/day for 48 weeks. The serum ALT level, HBsAg, HBeAg, anti-HBc, anti-HBe, anti-HBs and HBV DNA were tested every 12 weeks during the telbivudine therapy. Healthy donors (*n *= 10) were included as controls.

### Flow cytometry

Peripheral blood mononuclear cells (PBMC) were isolated from ethylenediaminetetraacetic acid (EDTA) anticoagulated blood samples on a Ficoll-Histopaque density gradient. After isolation, cells were washed twice in phosphate-buffered saline (PBS) and studied immediately. CD127, CD8, CD27 and CD45RA expression on the PBMC was detected by direct staining.

CD127 expression on HBV-specific CD8^+ ^T cells was performed as described previously [10]. Briefly, PBMC were stained with surface PC5-anti-CD8 (BD Pharmingen, San Diego, CA, USA) and pentamer+ CD8 T cells were detected by staining with phycoerythrin (PE)-labelled pentameric peptide-HLA2 complex (ProImmune, Oxford, UK) containing HBV Core 18-27 (FLPSDFFPSV) and HBV Core 18-27 (FLPSDFFPSI). Gated on CD8 T cells, CD127 expression on HBV-specific CD8 T cells was analyzed by fluorescein isothiocynate (FITC)-anti-CD127 and PE-labelled pentamers. Cells were washed three times with PBS, and 1 × 10^6 ^events in the lymphogate were collected by flow cytometry (EPICSXL; Coulter, Fullerton, CA, USA). Data were analyzed using CellQuest software (Coulter).

### Statistical analysis

The Wilcoxon matched pairs test and the Mann-Whitney test of SPSS version 12.0 were used to assess differences among groups. Spearman correlation analysis was performed between CD127 expression and serum HBV DNA and HBeAg levels. *P*-values less than 0.05 were considered statistically significant.

## Results

### CD127 expression on memory CD8 T cells was reduced in patients with chronic hepatitis B

**Figure 1:**
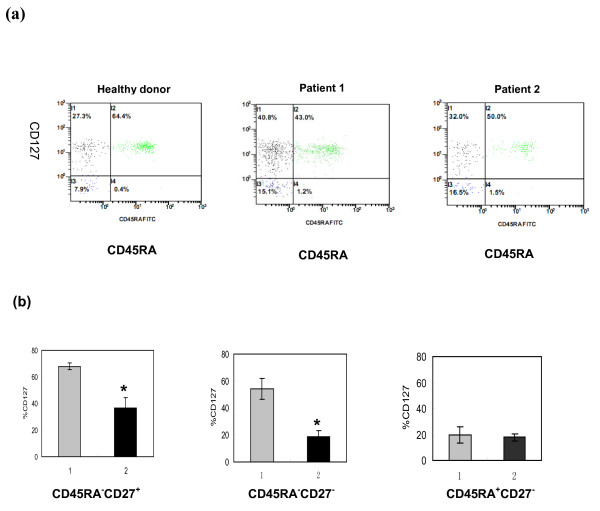
***Ex vivo *expression of CD127 by memory CD8 T cells taken from chronic hepatitis B (CHB) patients**. (a) CD127 expression by CD27^+^CD45RA^+ ^naive CD8 T cells. Results are shown for one healthy control and two CHB patients. (b) Proportion of CD127^+ ^lymphocyte cells in the memory (CD45RA^-^CD27^+^), effector (CD45RA^-^CD27^-^) and terminally differentiated effector (CD45RA^+^CD27^-^) CD8 T-cell subsets in healthy controls and CHB patients (*n *= 20 for each group). Healthy controls (gray bar); CHB patients(black bar). *P < 0.05 when compared to healthy controls.

*Ex vivo *expression of CD127 by different CD8 T lymphocyte subsets taken from patients with CHB were checked by flow cytometry. As indicated in Fig. 1a, naive CD8 T cells (CD45RA^+^CD27^+^) from CHB patients showed a high percentage of CD127^+ ^cells, as did the cells from healthy controls. When the expression of CD127 was examined in memory CD8 T cells (CD45RA^-^CD27^+^) and effector CD8 T cells (CD45^-^RACD27^-^), we found significant decrease of CD127 expression in CHB patients compared with healthy controls. Terminally differentiated effector CD8 T cells (CD45RA^+^CD27^-^) from both CHB patients and healthy controls expressed little CD127, as indicated in Fig. 1b.

**Figure 2:**
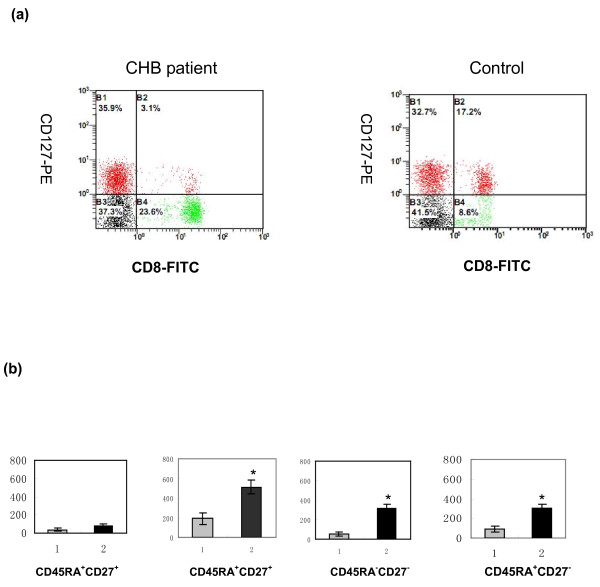
**Decreased CD127 expression on memory CD8 T cells from chronic hepatitis B (CHB) patients**. (a) Representative dot plots showing the expression of CD127 on CD8 T cells in one CHB patient and one healthy control. The numbers on the right indicate the percentages of CD8 T cells that are CD127^+ ^and CD127^-^. (b) Absolute counts per cubic millimetre of total CD127^- ^lymphocytes in naive, memory, effector and terminally differentiated CD8 lymphocytes from each study group. Healthy controls (gray bar); CHB patients(black bar). *P < 0.05 when compared to healthy controls.

To determine whether the decreased percentage of CD8^+^CD127^+ ^memory T cells reflected an absolute reduction of these cells or increase of CD8^+^CD127^- ^memory T cells, we calculated the absolute counts of CD127^+ ^and CD127^- ^memory cells in the total CD8 T cells as well as in the naive- and memory-cell subsets. As indicated in Fig. 2a, the absolute number of CD8^+ ^T cells expressing CD127 was similar in CHB patients and in healthy controls. But the absolute number of CD8^+^CD127^- ^T cells increased significantly in CHB patients compared with healthy controls. Importantly, the absolute CD127^- ^memory T-cell count increased markedly in CHB patients as well, while only a small increase in the number of CD127^- ^naive T cells was detected in the same group of patients (Fig. 2b). These results imply that HBV infection is associated with a marked up-regulation of memory T cells that have decreased expression of CD127.

### Relationship between CD127 expression on memory CD8 T cells and serum HBV DNA and HBeAg levels in patients with chronic hepatitis B

**Figure 3:**
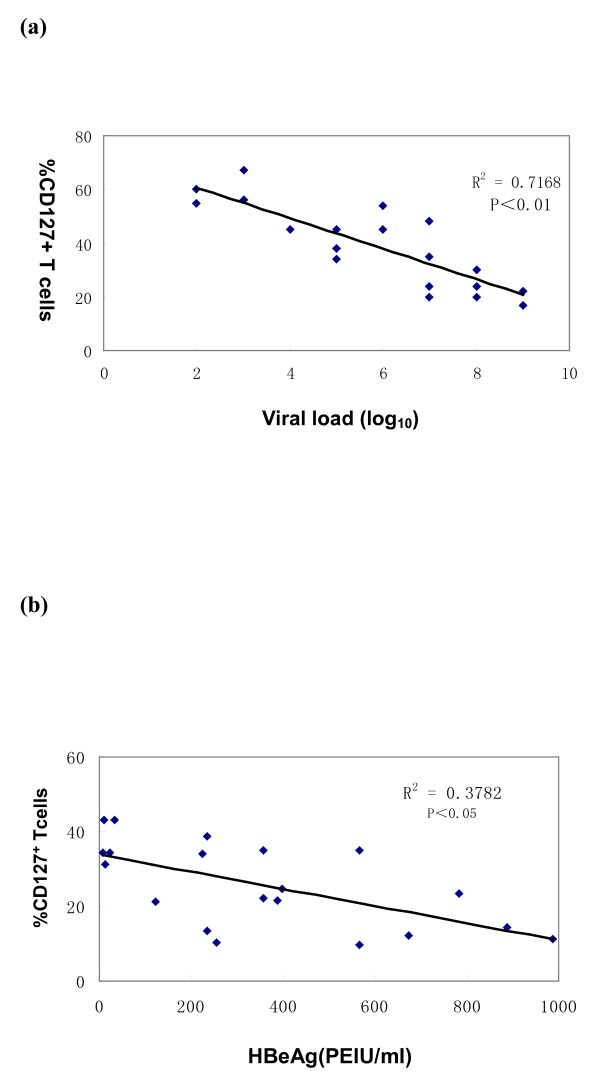
**The relationship between CD127 expression on memory CD8 T cells and serum hepatitis B virus (HBV) DNA and hepatitis B e antigen (HBeAg) levels in chronic hepatitis B (CHB) patients**. (a) Expansion of CD127^+ ^memory CD8 T cells is correlated inversely with serum HBV DNA level in CHB patients. (b) Expansion of CD127^+ ^memory CD8 T cells is correlated inversely with serum HBeAg level in CHB patients. All analyses were performed on the 20 CHB patients described in the Materials and Methods.

We next investigated a possible relationship between the CD127 expression of CD8 memory T cells of CHB patients and the main viral markers of HBV infection: serum HBV DNA and HBeAg. There was a strong negative correlation between CD127 expression on CD8 memory T cells and these markers in CHB patients, as indicated in Fig. 3.

### Antiviral therapy increased CD127 expression on CD8 memory T cells in patients with chronic hepatitis B

**Figure 4:**
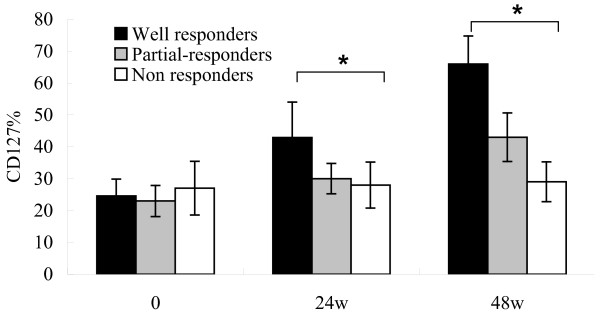
**Antiviral therapy increases CD127 expression on CD8 memory T cells in chronic hepatitis B (CHB) patients**. The mean expression of CD127 on CD8 memory T cells. The analysis was performed on 20 CHB patients treated with telbivudine at 0, 24 and 48 weeks. Statistical analyses were performed among 6 well responders (black bar), 10 partial responders (gray bar) and 4 non responders (white bar) (*P < 0.05).

We selected 20 HBeAg-positive CHB patients who were treated with telbivudine 600 mg/day for 48 weeks. After 48 weeks of treatment, 6 patients became HBV DNA negative by PCR assay and had HBeAg seroconversion. These six patients were defined as 'well responders'. Four patients whose serum HBV DNA levels remained at more than 5 log_10 _copies/mL and HBeAg remained positive at Week 48 were defined as 'non-responders'. The other ten patients were defined as 'partial responders'. We dynamically compared the proportion of CD127 expression on the memory CD8 T cells among the well responders, partial responders and non-responders. As indicated in Fig. 4, telbivudine significantly increased CD127 expression on memory CD8 T cells from well responders compared with non-responders (*P *< 0.05).

### Emergence of CD127 HBV-specific CD8^+ ^T cells after successful antiviral treatment

**Figure 5:**
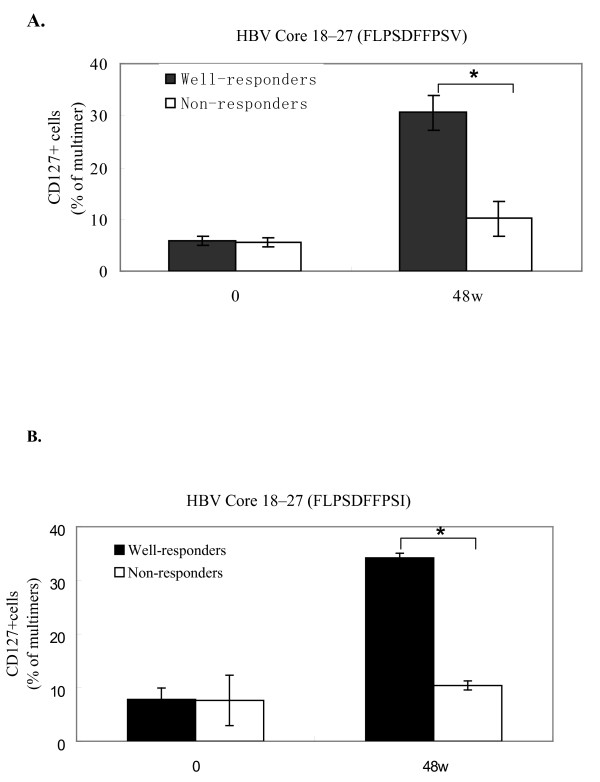
**The emergence of CD127 hepatitis B virus (HBV)-specific CD8^+ ^T cells after successful antiviral treatment**. Surface staining of HBV-specific CD8 T cells was performed using a HBV multimer [**A**. HBV Core 18-27 (FLPSDFFPSV), **B**. HBV Core 18-27 (FLPSDFFPSI)] and antibody to CD127, as described in the Materials and Methods. Data are shown as the percentage of multimer-positive CD8 T cells from 6 well responders (black bar) and 4 non responders (white bar) of CHB patients treated with telbivudine at 0 and 48 weeks respectively. (*P < 0.05).

We compared the expression of CD127 on HBV-specific CD8^+ ^T cells from CHB patients before and after telbivudine treatment. As indicated in Fig. 5A and 5B, CD127 expression increased markedly in HBV-specific CD8 T cells in telbivudine responders compared with non-responders (*P *< 0.05).

## Discussion

In our study, we demonstrated that CD127 expression on memory CD8 T cells was reduced in patients with CHB. There was a strong negative correlation between CD127 expression on memory CD8 T cells and serum HBV DNA and HBeAg levels in CHB patients. Moreover, successful antiviral therapy with telbivudine increased CD127 expression on CD8 memory T cells as well as on HBV-specific CD8 T cells in CHB patients.

It is widely accepted that CD8 T cells play an essential role in the immune response to viral infection. In successful responses to acute HBV, hepatitis C virus (HCV) and lymphocytic choriomeningitis virus (LCMV) infection, the up-regulation of CD127 expression on CD8 T cells is closely associated with the downregulation of CD38 and PD-1 and the upregulation of CCR7 expression [9,11,12]. All occurred in concert with resolution of disease and containment of viral antigen, supporting the theory that the emergence of CD127 is governed by withdrawal of antigenic stimulation. Colle *et al. *[13] reported that human immunodeficiency virus (HIV) infection was associated with a decrease in the proportion of CD127^+ ^cells among memory CD8 T lymphocytes, which resulted in a higher CD127^- ^CD8 T cells count in patients with HIV infection. There was a strong negative correlation between CD127 expression on CD8 T cells and HIV viral load [14]. All of these results support the hypothesis that high CD127 expression on human CD8 T cells is specific for cleared virus [e.g. influenza virus, respiratory syncytial virus (RSV) and acute HBV infection] while low CD127 expression on human CD8 T cells is specific for persisting virus [e.g. HIV, cytomegalovirus (CMV), HCV and HBV] [15].

Recently some reports have suggested that CD127 might be a useful marker for predicting response to antiviral therapy in HIV- and HCV-infected patients. Badr *et al. *[11] reported that during HCV infection, early therapeutic intervention with pegylated (PEG)-interferon (IFN)-α rescued long-lived, polyfunctional memory CD8 T cells expressing high levels of CD127 and Bcl-2 (CD127^hi^Bcl^hi^). In contrast, HCV-specific CD8 T cells in acute infections evolving to chronicity expressed low levels of CD127 and Bcl-2, exhibited diminished proliferation and cytokine production, and eventfully disappeared from the periphery. Colle *et al. *[13] also reported that CD127 was increased in memory CD8 T lymphocytes from HAART patients. Our longitudinal study indicated that successful antiviral therapy with telbivudine increased CD127 expression on CD8 memory T cells as well as on HBV-specific CD8 T cells in CHB patients. These consistent results clearly suggest that measurement of CD127 expression might be useful for predicting response to antiviral therapy.

In chronic HBV-infected patients, the frequency and function of circulating and intrahepatic antiviral T-cell responses is inversely proportional to the level of HBV DNA [16,17]. Nucleoside analogues are known to interfere with viral replication, directly lowering HBV DNA levels, but whether they influence the development of effective memory T-cell differentiation and function has not been proven. Our findings indicate that treatment-induced suppression of HBV replication resulted in upregulation of CD127 expression on memory CD8 T cells in all well responders to telbivudine, but not in non-responders. These comparison results obtained in the responders and non-responders to antiviral therapy support the notion that increased expression of CD127 on memory CD8 T cells is linked to successful inhibition of viraemia. These results indicate measurement of CD127 expression on memory CD8 T cells may be useful to guide antiviral therapy in patients with CHB. However, longitudinal studies are required to draw a clear conclusion on this matter.

Taken together, our results suggest the mechanism linking HBV replication and abnormalities in CD8 T-cell function in patients with CHB. We also demonstrate a strong negative correlation between HBV viraemia and CD127 expression in memory CD8 T cells. Telbivudine-induced inhibition of HBV replication resulted in significant upregulation of CD127 expression in memory CD8 T cells, reducing its negative influence on CD8 T cells' activation and function in CHB patients. Most important, we demonstrate successful antiviral treatment can rescue such a functional signature on memory CD8 T cells, which will indicate to achieve sustained inhibition of HBV replication and resolution of chronic liver disease [18].

## Competing interests

The authors declare that they have no competing interests.

## Authors' contributions

LGC and YLJ performed the majority of experiments and contributed equally to this work. MWJ did most of clinical works. JX and ZL provided analytical tools and were also involved in editing the manuscript, YYD designed the study and wrote the manuscript.
